# SnakeWRAP: a Snakemake workflow to facilitate automated processing of metagenomic data through the metaWRAP pipeline

**DOI:** 10.12688/f1000research.108835.2

**Published:** 2022-04-28

**Authors:** John Krapohl, Brett E. Pickett

**Affiliations:** 1 Department of Microbiology and Molecular Biology, Brigham Young University, Provo, UT, 84602, USA

**Keywords:** metaWRAP, shotgun metagenomics, high-performance computing, big data, Snakemake

## Abstract

Generating high-quality genome assemblies of complex microbial populations from shotgun metagenomics data is often a manually intensive task involving many computational steps. SnakeWRAP is a novel tool, implemented in the Snakemake workflow language, to automate multiple metaWRAP modules. Specifically, it wraps the shell scripts provided within the original metaWRAP software, within Snakemake. This approach enables high-throughput simultaneous assembly and analysis of multiple shotgun metagenomic datasets using the robust modular metaWRAP software. We expect this advancement to be of import in institutions where high-performance computing infrastructure is available, especially in the context of big data. This software tool is publicly available at https://github.com/jkrapohl/SnakeWRAP

## Introduction

As sequencing technology has become cheaper and more readily accessible, the need for the increased computational capacity to process these data has become apparent. In particular, high-throughput sequencing has been particularly useful when applied to the field of metagenomics. Substantial effort has been devoted to developing software and computational pipelines, such as MetaWRAP, which cater to this growing area of research. MetaWRAP is a modular wrapper that combines many of the necessary tools to process reads, create bins, and visualize data within a robust modular design ( Uritskiy, DiRuggiero, & Taylor, 2018). The primary limitation arising from this design is the inability to automatically scale its usage to massive datasets. However, its unique code that produces exceptionally high quality bins from bins generated from other binning programs is invaluable. Snakemake is a widely-used Python based workflow management system that automates repetitive tasks, allowing software processes to be both scalable and reproducible ( Mölder *et al.*, 2021). By integrating the original MetaWRAP processes into Snakemake, the customizable modular nature of MetaWRAP can be preserved even when automatically processing large datasets through a single workflow. Our SnakeWRAP software automates the tasks performed within MetaWRAP, allowing for individual modules to be toggled on and off using Snakemake-defined rules.

## Methods

### Implementation

As this tool makes use of Snakemake, individual steps of the workflow are broken into rules, many of which can be toggled ( Mölder *et al.*, 2021). To do this, Snakemake requires a YAML configuration file as an input to determine which steps are specified for the run, as well detailed parameters such as which assembly tool to use. This configuration file also specifies the location of a metadata file, which contains a list of files associated with the run. An example of both the YAML and metadata file are included in the Github repository. SnakeWRAP can be submitted for scheduling from the command line by using the submission script also found within the repository. Running the script requires the complete installation of both Snakemake and METAWrap, as well as all of their dependencies. It is recommended that Snakemake and METAWrap be installed using the latest version of Miniconda, following instructions found on their corresponding Github repositories ( Mölder *et al.*, 2021; Orjuela, Huang, Hembach, Robinson, & Soneson, 2019).

### Operation

The advantage of using SnakeWRAP over the base METAWrap program is the ability to appropriately support large-scale job sizes while requiring less user input by automating the entire process. This enables jobs to be better organized and allows improved consistency in outputs. We recommended that this tool be run on cluster computing or within a high-performance infrastructure for larger jobs. In such cases, we have found that a minimum of 100 GB of RAM across 24 CPUs is sufficient, with larger jobs requiring additional resources.

The rules found within the Snakemake file correspond to different steps within the original METAWrap software. Some initial required steps include read quality control, assembly, binning, and bin refinement and reassembly. However, we have provided toggle settings for memory-heavy modules that generate figures, which are Blobology, Kraken, bin quantification, bin annotation, and bin classification. This design reduces resource usage by only generating the desired figures, and allows the modification of this setting to create these figures at a later time by toggling the module on in the YAML file, deleting any outputs created for that module, and resubmitting the job. Snakemake will skip any rules already completed and only run the required rule(s).

### Use cases

As stated above, this tool works best with a high-performance computing infrastructure. We anticipate that the most effective use of this tool is applying it to big metagenomics data, such as meta-analyses of existing datasets or datasets with many samples. To assess the capabilities of this tool, the publicly available samples SRR13296364 and SRR13296365, accessed from the NCBI Sequence Read Archive from the study SRP299130, were run together successfully. We then ran a larger set of files from the SRP257563 study through the metaWRAP pipeline using this software. We found that this tool will run as expected as long as sufficient memory is provided and all dependencies are installed correctly. Inputs include sequencing files in fastq format, a metadata file listing all fastq filenames, and a YAML file describing paths for all file sources and destinations. Outputs include high-quality genomic bins, as wells as a number of generated figures such as a blobplot, heatmap, and kronogram.

## Discussion

While the processing of metagenomics datasets is untractable for most personal computers, researchers with access to high-performance computing infrastructure can take full advantage of this software. The core functions found within the original MetaWRAP include read quality control, assembly, and binning are required for the analysis and cannot be toggled off. These functions include several refinement steps that are unique to MetaWRAP, which allow it to create higher-quality bins than existing stand-alone programs ( Uritskiy *et al.*, 2018). Our design enables the user to decide whether computationally-expensive modules, such as Kraken and Blobology, that assist with figure generation can be skipped.

Snakemake automatically generates a directed acyclic graph (DAG) to order tasks, track the progress of each task for each sample, and eliminate duplicate tasks for the same sample ( Mölder *et al.*, 2021). This is vital to efficiently processing and analyzing large datasets, as jobs can fail due to insufficient memory or timing out. An advantage of implementing this workflow in Snakemake is the ability to resume the workflow from the same step that was underway if a process fails. The input paths, output paths, and parameters for each job are assigned by the end-user within a configuration file. This file is read by Snakemake to prevent data from being incorrectly assigned or lost and to facilitate reuse and customization of the workflow. We anticipate that this advance in automating metagenomics data processing will facilitate the generation, analysis, and re-analysis of larger datasets in the future.

## Data availability

No data are associated with this article.

## Software availability

Source code available from: https://github.com/jkrapohl/SnakeWRAP.

Archived DOI at time of publication: https://doi.org/10.5281/zenodo.5719960.

License: Open.
