# Supplementary material for: SnakeWRAP: a Snakemake workflow to facilitate automated processing of metagenomic data through the metaWRAP pipeline
Source: F1000Res. 2022 Apr 28;11:265. Originally published 2022 Mar 2. [Version 2] doi: 10.12688/f1000research.108835.2 (PMC10352622; doi:10.12688/f1000research.108835.2)
Supplement: Peer review history

# Peer review: Reviewer response for version 2

**DOI:** 10.5256/f1000research.133574.r186472

**Published:** 2023-07-18

**Authors:** Lauren Mak

**Affiliations:** 1 Weill Cornell Medicine, New York, USA

The authors have developed a Snakemake wrapper around MetaWRAP modules to address two structural shortcomings in the original pipeline: 1) Its inability to take advantage of HPC resources and 2) its inability to switch resource-intensive steps on or off. The authors successfully used Snakemake to address these pain points by encapsulating each module and letting the Snakemake scheduler manage resource allocation and option ingestion. Furthermore, because MetaWRAP is no longer being maintained, some kind of updated structure is needed to manage the analysis of large datasets.

 While the authors have indeed addressed the pain points described, they have made some software architecture choices that make the utility of their pipeline questionable.

**Major:**

 For one, their installation process involves installing MetaWRAP and SnakeWRAP separately. An more efficient way around this would be to fork MetaWRAP and integrate their SnakeWRAP code, thereby obviating the need to add a MetaWRAP path to the config. However, this might not even be the best option - MetaWRAP has many documented environment management issues, which is to be expected given the pace of the field. How does SnakeWRAP handle this? The Snakefile doesn't seem to have conda integration. 

 Re: '...sufficient information provided to allow interpretation of the expected output...' and '...conclusions about the tool and its performance adequately supported'. No explanation at all, not even a summary of the original MetaWRAP output. Furthermore, there is no technical comparison (ex. in terms of runtime, at least) to support the claim that the pipeline is a more efficient implementation of MetaWRAP. 

 Re: '...sufficient details of the code... to allow replication...' In the README, some installation commands are provided, though instructions on how to download the FastQs from the SRA are missing. Furthermore, there is no `metatext.txt` for samples, only `sample_metadata.txt` which contains not FastQ but SRA pathnames. 

**Minor:**

 While the number of threads is handled by the config, it seems the amount of memory allocated to each job is not. From the Slurm submission script, it appears that the mem-per-cpu flag allocates a certain amount per thread. Is this necessarily the best setup? What if a task parallelizes poorly but requires the storage of a large matrix in memory (i.e.: anything loading a database)? 

 A purely philosophical question - why are Bash scripts called in the Snakefile, when the purpose of the shell block is to run commands Bash-style?

Are the conclusions about the tool and its performance adequately supported by the findings presented in the article?

Partly

Is the rationale for developing the new software tool clearly explained?

Yes

Is the description of the software tool technically sound?

Yes

Are sufficient details of the code, methods and analysis (if applicable) provided to allow replication of the software development and its use by others?

Partly

Is sufficient information provided to allow interpretation of the expected output datasets and any results generated using the tool?

Partly

Reviewer Expertise:

Metagenomics analysis, pipeline development, strain inference, MAG binning, assembly graphs, evolutionary biology

I confirm that I have read this submission and believe that I have an appropriate level of expertise to confirm that it is of an acceptable scientific standard, however I have significant reservations, as outlined above.

# Peer review: Reviewer response for version 2

**DOI:** 10.5256/f1000research.133574.r186487

**Published:** 2023-07-17

**Authors:** Michael J Roach

**Affiliations:** 1 Flinders University, Adelaide, Australia

The authors describe a Snakemake pipeline that automates running the popular metaWRAP pipeline. The metaWRAP bash scripts that SnakeWRAP runs are themselves bash pipelines that run external programs and perform intermediate steps. It would be far more beneficial to perform a more complete rewrite of the metaWRAP package entirely within the Snakemake framework. Looking at the metaWRAP flowcharts, I can appreciate that this would be a much bigger task. My main suggestion otherwise would be to ship the pipeline with a tiny test dataset that the users can run, and to provide somewhere (zenodo perhaps) the example outputs that the authors allude to in the manuscript. 

**Other thoughts:**

- I would recommend a more meaningful name. There are thousands of SNAKEmake pipelines and most of them are WRAPpers for external tools.
- Use standardised structure for snakemake pipelines: https://snakemake.readthedocs.io/en/stable/snakefiles/deployment.html#distribution-and-reproducibility
- Consider including snakemake rules to download databases and metaWRAP.
- You can read in a config file within the Snakefile using the "configfile:" directive. This will initialise config with sensible defaults, but any user-supplied config (either modified in the default config file, or in a user-supplied config file, or passed with -C) will overwrite these defaults. This will save you having to initialise empty config settings and sanitise filepaths at the start.
- Consider alternate target rules to rule all, e.g. rule qc could specify just the qc module which the users could run with "snakemake...qc". This could potentially replace the config toggles, e.g. the user would run "snakemake...assembly kraken prokka" and the stages not specified (blobplot/salmon/etc) would not be run. 
- Specify resources for use with snakemake profiles on HPC clusters etc. These can be parsed from the config file.
- Consider messages instead of echo in shell commands: https://snakemake.readthedocs.io/en/stable/snakefiles/rules.html#messages
- You can move rules to one or more external files and add them in with the "include:" directive if your Snakefile is getting too big.
- You can use "script:" instead of "shell:" for your bash scripts: https://snakemake.readthedocs.io/en/stable/snakefiles/rules.html#bash

Are the conclusions about the tool and its performance adequately supported by the findings presented in the article?

Partly

Is the rationale for developing the new software tool clearly explained?

Yes

Is the description of the software tool technically sound?

Yes

Are sufficient details of the code, methods and analysis (if applicable) provided to allow replication of the software development and its use by others?

Yes

Is sufficient information provided to allow interpretation of the expected output datasets and any results generated using the tool?

Yes

Reviewer Expertise:

Bioinformatics; genomics; metagenomics

I confirm that I have read this submission and believe that I have an appropriate level of expertise to confirm that it is of an acceptable scientific standard.

# Peer review: Reviewer response for version 1

**DOI:** 10.5256/f1000research.120268.r126391

**Published:** 2022-03-29

**Authors:** Tobias Jakobi

**Affiliations:** 1 Department of Internal Medicine and the Center for Translational Cardiovascular Research, University of Arizona, Phoenix, AZ, USA

The manuscript submitted by Krapohl *et al.* outlines a Snakemake-based framework for the metaWRAP software. Given complex workflows like metaWRAP it makes sense to streamline operations by using workflow managers such as Snakemake. The authors provide a good overview of the feature of their software, but the addition of more details in different parts of the manuscript might help readers and potential users to get a better picture.

1. The abstract mentions the metaWRAP software, but does not really introduce what metaWRAP does. Although there is clearly a connections to metagenomics, one sentence to introduce the software would make the link more direct.
2. The introduction only focuses on metaWRAP; here, mentioning other, similar tools would make the introduction more inclusive. Why did the authors choose metaWRAP as basis for their software and not another tool?
3. The snakemake-defined rules probably do not require quotes.
4. Yaml should be capitalized to YAML and spelled out when it’s first used.
5. The authors state that 100GB RAM and 24 CPUs are sufficient for “normal” jobs. Here, it would be very helpful to give readers and potentials user an idea what “normal” and “larger” jobs correspond to, e.g. number of reads, or libraries?
6. The authors refer to the suitability of the approach for HPC environment. However, are any interfaces to specific scheduling systems included? The most time consuming step of setting up new HPC pipelines usually is the scheduling setup.
7. The authors write that two different datasets completed successfully, but how long did the run take? Of course that’s dependent on the hardware used, but it should be quantifiable.
8. There seems is another, unrelated software on GitHub that uses the same name: https://github.com/EthanHolleman/metasnake.

Are the conclusions about the tool and its performance adequately supported by the findings presented in the article?

Partly

Is the rationale for developing the new software tool clearly explained?

Yes

Is the description of the software tool technically sound?

Yes

Are sufficient details of the code, methods and analysis (if applicable) provided to allow replication of the software development and its use by others?

Yes

Is sufficient information provided to allow interpretation of the expected output datasets and any results generated using the tool?

Yes

Reviewer Expertise:

Bioinformatics, High Performance Computing, Computational RNA Biology, Cardiovascular Disease

I confirm that I have read this submission and believe that I have an appropriate level of expertise to confirm that it is of an acceptable scientific standard.
